# A study on biological activity of marine fungi from different habitats in coastal regions

**DOI:** 10.1186/s40064-016-3658-3

**Published:** 2016-11-14

**Authors:** Songlin Zhou, Min Wang, Qi Feng, Yingying Lin, Huange Zhao

**Affiliations:** 1 Key Laboratory of Tropical Diseases and Translational Medicine of The Ministry of Education, Hainan Medical College, Haikou, 571199 China; 2 Department of Aquatic Science and Technology, Jiangsu Agri-animal Husbandry Vocational College, Taizhou, 225300 China

**Keywords:** Marine fungus, Habitats, Diversity, Biological activity

## Abstract

In recent years, marine fungi have become an important source of active marine natural products. Former researches are limited in habitats selection of fungi with bioactive compounds. In this paper were to measure antibacterial and antitumor cell activity for secondary metabolites of marine fungi, which were isolated from different habitats in coastal regions. 195 strains of marine fungi were isolated and purified from three different habitats. They biologically active experiment results showed that fungi isolation from the mangrove habitats had stronger antibacterial activity than others, and the stains isolated from the estuarial habitats had the least antibacterial activity. However, the strains separated from beach habitats strongly inhibited tumor cell proliferation in vitro, and fungi of mangrove forest habitats had the weakest activity of inhibiting tumor. Meanwhile, 195 fungal strains belonged to 46 families, 84 genera, 142 species and also showed 137 different types of activity combinations by analyzing the inhibitory activity of the metabolites fungi for 4 strains of pathogenic bacteria and B-16 cells. The study investigated the biological activity of marine fungi isolated from different habitats in Haikou coastal regions. The results help us to understand bioactive metabolites of marine fungi from different habitats, and how to selected biological activity fungi from various marine habitats effectively.

**Electronic supplementary material:**

The online version of this article (doi:10.1186/s40064-016-3658-3) contains supplementary material, which is available to authorized users.

## Background

Marine habitats can be categorized into two types: coastal region habitats, deep-sea region habitats (Richards et al. 2012). Coastal regions are characterized by eutrophication from terrestrial run-off and high primary production, on the other hand this leads to large availability of organic matter to consumers as detritus (Danovaro and Pusceddu 2007). Marine fungi are known to play an important role as primary degraders in coastal waters (Massana and Logares 2013). Due to the long coastal line, therefore, a lot of unique niches were found such as the coral reef habitats, the mangrove habitats, estuarine habitats, beach habitats, rocky habitats etc.(Richards et al. 2012; Rämä et al. 2014). There are many reports either focusing on the use of molecular techniques or the traditional taxonomic methods to investigate microbial diversity from marine environments (Miloslavich et al. 2010; Singh et al. 2012; Klindworth et al. 2014), or reports researching marine fungi isolated from all kinds of new compounds with various biological activities in different habitats (Blunt et al. 2014). However, few studies reported the active marine fungi interrelation between the biological activity of metabolites and separate from habitats. In this study we examined the antibacterial and antitumor activity of fungi isolated from three different coastal niches, which can not only enriched the diversity of marine fungus, but also helped select antibacterial or anti-tumor active strains.

Haikou is located in the tropical northern margin of low latitude, which has a tropical oceanic monsoon climate. Coastal areas have several niches, such as beach habitats, rocky shoreline habitats, mangrove habitats. These habitats were selected for which may display extreme abundance in microbial biodiversity.

## Methods

### Cell lines and pathogenic bacteria

B16 Melanoma cells, *Staphlococcus aureus* ATCC25923, *Pseudomonas aeruginosa* ATCC 27853, *Klebsiella pneumoniae* ATCC700603 and *Escherichia coli* ATCC 25922 were provided by the National Institute for the Control of Pharmaceutical and Biological Products (Beijing, China).

### Sites and sample collection

**Fig. 1:**
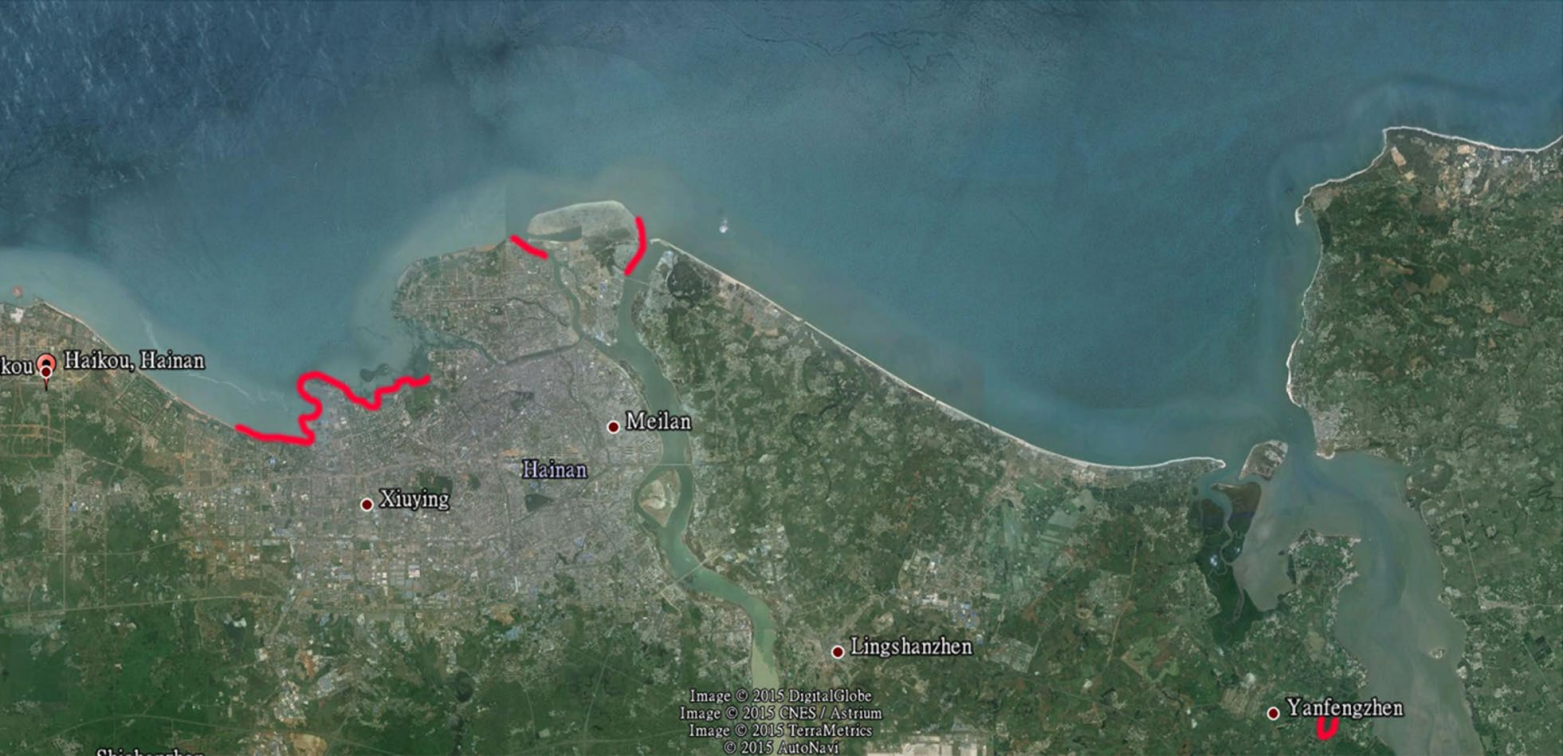
Red tag areas indicated the location of sample sites

Seawater, sediments and rotted leaves were collected from three coastal marine habitats (beach habitats of west coastal Haikou, estuarine habitats of Nan Dujiang River, and mangrove habitats of YanFeng town) in Haikou during March, 2012 (Fig. 1 and Table 1). These samples were carried in sterile, screw capped plastic bottles. The isolation of fungi from the seawater samples was finished using the membrane filtration technique within one hour after collection. 15 ml water samples were filtered through sterile 0.45 μm in cellulose ester membranes. These membranes were then placed on the ordinary separation medium (Glucose 10 g/L, peptone 2 g/L, yeast extract 1 g/L, agar 20 g/L, streptomycin 75 mg/L, ampicillin 50 mg/L, aged seawater 600 mL/L). Sediment samples (0.1 g) were suspended in 10 ml sterile seawater and 100 μl of the resulting suspension was plated directly on the ordinary separation medium.

**Table 1:** Details regarding the sample types and geographical distribution and isolated fungal colonies

Sampling date	Location	Lat (°N)	Long (°E)	Habitat	Depth (m)	Temperature (°C)	Salinity (ppt)	No. of samples	No. of stains
2012-3-18	West Coastal of Haikou	20°1′53″–1′3″	110°18′17″–14′23″	Seawater	0	21.7	31.7	7	13
				Sediments	0	21.7	31.7	7	11
				Rotted leaves	0	21.7	31.7	30	82
2012-3-22	Nan Dujiang River	20°4′48″–4′2″ 20°4′36″–4′21″	110°22′43″–22′30″ 110°20′26″–20′56″	Seawater	0	23.3	20.1~23.5	5	8
				Sediments	0	23.3	20.1~23.5	5	5
				Rotted leaves	0	23.3	20.1~23.5	10	23
2012-3-28	Yan Feng Mangrove	19°57′1″–57′11″	110°34′53″–35′27	Seawater	0	20.6	19.6	4	8
				Sediments	0	20.6	19.6	4	7
				Rotted leaves	0	20.6	19.6	12	38

Rotted leaves were washed thoroughly three times with sterile distilled water, surface sterilized with 5% NaClO for 5 min, 75% ethanol for 1 min, and sterile distilled water twice. Each sample was then cut into 4 mm to 6 mm sections and placed on ordinary separation medium plates. All plates were incubated at 25 °C and examined daily for the growth of fungi. Fungal colonies that developed were sub-cultured onto fresh ordinary separation medium plate for pure, single colony isolation and identification. The identification of filamentous fungi was done by macroscopic and microscopic morphology.

### DNA extraction, amplification and sequencing

The chromosomal DNA from all strains were extracted using a fungus genomic DNA extraction kit (Tiangen Co., China), and the isolated DNA was amplified by PCR using universal primers ITS 1 and ITS 4 (Rivera and Seifert 2011), corresponding to a 5.8S rDNA sequence. For PCR, the reaction elements included 1 μL of the template DNA, 5 μL of 10× Taq buffer, 1 μL of 10 mM mixed dNTP, 1 μL of sense-ITS 1 primer (TCCGTAGGTGAACCTGCGG, 10 pmol/μL), 1 μL of antisense-ITS 4 primer (TCCTCCGCTTATTGATATGC, 10 pmol/μL), and 5 U of Taq DNA polymerase, which were prepared in a final volume of 50 μL. The PCR thermal conditions were set as follows: preheating for denaturation at 94 °C for 4 min, 30 cycles of denaturation at 94 °C for 30 s, annealing at 52 °C for 30 s, extension at 72 °C for 60 s, and additional extension at 72 °C for 7 min. The PCR products were purified using Gel DNA extraction kit (NewTopBio, China). The sequencing analysis data obtained from Sinogenomax Co., Ltd. to blasting other sequences from GenBank comprising with identity sequences.

### Phylogenetic analyses

Forward and reverse sequences were edited and assembled using Chromas Pro version 1.34 (Technelysium Pty Ltd, Tewantia, Queensland, Australia). The final sequences were compared to the nucleotide sequences of reference organisms available in the GenBank database using Blastn. The ITS1-5.8S-ITS4 gene sequences obtained for the organisms were aligned with their closest match using the program ClustalW2. Gaps and ambiguously aligned sequences were removed manually from further analyses. Phylogenetic analyses were carried out using distance setting (Maximum parsimony) in MEGA 5 software with 1000 bootstrap replicates.

### Preparation of marine Fungal Extract

Each fungus was inoculated into a 500 mL Erlenmeyer flask containing 100 mL of liquid culture medium (which is general separation medium without penicillin and kalamycin). All shake flasks were incubated on a rotary shaker at 150 rpm, 25 °C for 20 d. Each fungal broth was separated into mycelia mat and culture filtrate by Whatman No. 1 filter paper. The mycelia mat was dried and grinded then extracted twice with acetone by ultrasonic condition. Subsequently, the filtrate was evaporated under reduced pressure (8 × 10^3^ Pa) to yield an extract and then extracted twice with acetone. Then merged all acetone soln. was concentrated to afford a crude extract.

### MTT assay

B-16 cell lines were grown in RPMI-1640 supplemented with 10% FBS (fetal bovine serum) in a humidified incubator at 37 °C with 5% CO2. Cell suspensions (190 μL, 5 × 10^4^ cell/mL) were plated in 96-well microtiter plates and incubated for 18 h. Then, different concentrations of the fungal metabolite extracts were added to each well and further incubated for 48 h. Then the MTT solution (20 μL; 5 mg/mL) was added to each well and incubated for 4 h. Old medium containing MTT was then gently replaced by DMSO (150 μL) and pipetted to dissolve any formazan crystals formed. Absorbance was then determined at 540 nm with a Spectra-Max-Plus plate reader.

### Antimicrobial test

Antimicrobial activity of the secondary metabolites from marine microorganisms was carried out by the K–B disk diffusion method (Drew et al. 1972). 0.2 mL of test bacterial solution, which was activated and diluted to 1/2 Mcfar turbidity, was evenly coated and inoculated in sterile Luria–Bertani (LB) broth agar, the drug disks (50 μg) were pasted on the inoculated agar plate, with four equidistant disks per plate, but one of them contained no drugs, and each test had 3 parallels. They were inverted in culture incubator at 37 °C and taken out after 18 h, the diameter of each inhibition zone was measured with vernier caliper, all assays was measured three times and averaged (n = 3).

### Statistical analysis

**Fig. 2:**
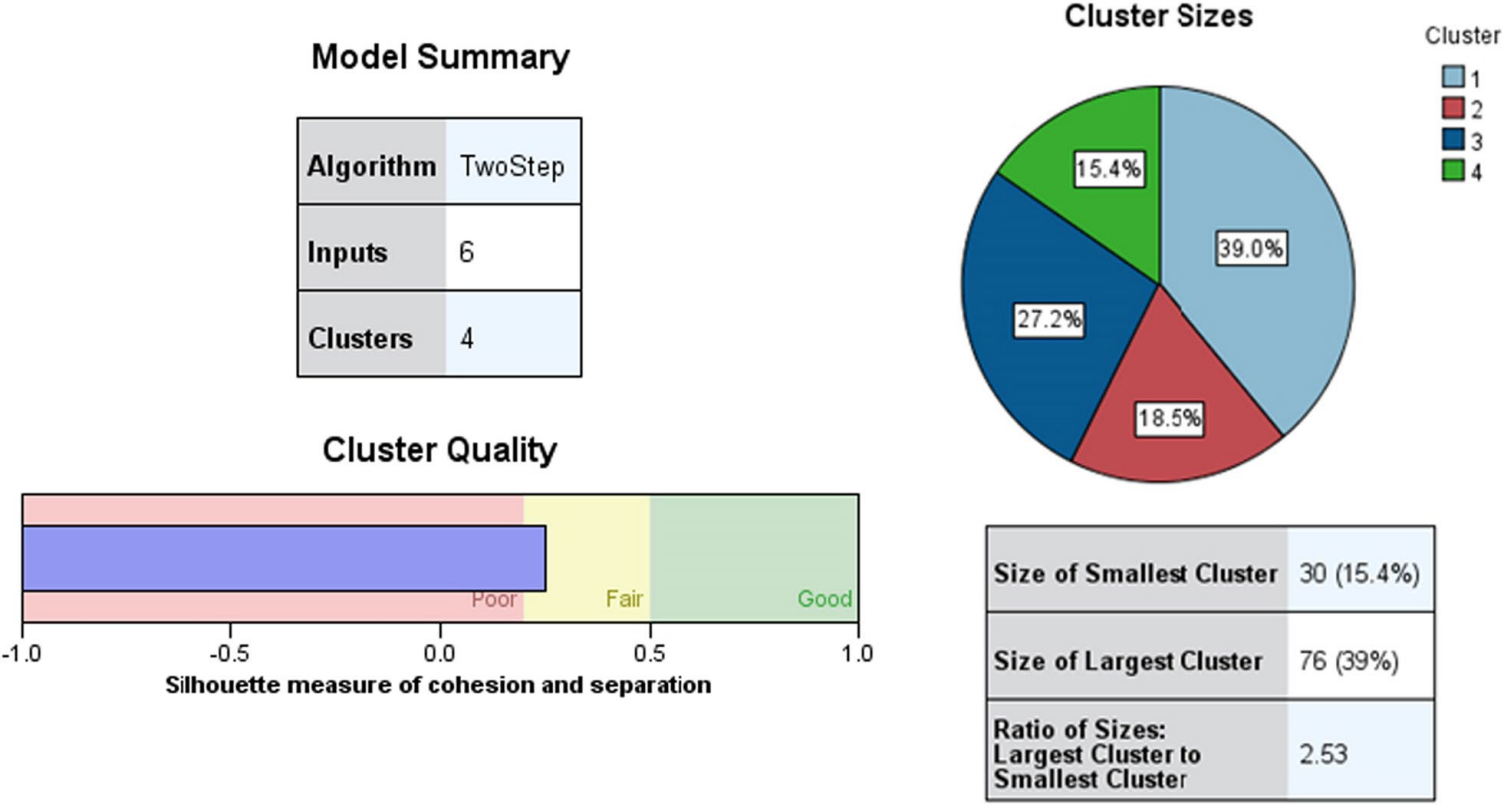
Three Habitats stains was isolated four Cluster by SPSS 22.0 using two step cluster analysis

One objective of this research was to identify various groups of fungi according to their habitats and biological activity, and therefore the two step cluster analysis was performed using SPSS 22.0 (Fig. 2). Before data analysis, the raw data were transformed. Three habitats were denoted by numbers (Beach habitats-1, Estuarine habitats-2, Mangrove habitats-3). The inhibitory effects were converted to numbers (“−” 0, “+” 1, “++” 2, “+++” 3) and then transformed into Z scores. In cluster analysis, habitats were selected as categorical variables, the inhibitory effects against different bacterial strains and tumor cells were classified into continuous variables. After that, the frequency of different combination of inhibitory effects was described with Excel data explorer using transformed data.

## Results

### Culturable diversity

A total of 195 marine fungal strains were found in 73 samples from Haikou coastal regions. Isolations from various habitats were shown in Table 1. 106 strains of fungi were isolated from the west coast of Haikou (designated as XB-1 to XB-106); 36 strains from the Nandu River Estuary habitats (named after NE-1 to NE-36); and 53 strains from the YanFeng Town mangrove habitats (labeled with YM-1 to YM-53).

195 marine fungal strains belonged to 46 families, 84 genera, 142 species, the strains mostly belonged to Ascomycota, Basidiomycota and Zygomycota based on ITS rRNA gene analysis (Additional file 1: Table S1; Additional file 3: Figure S1), The Aspergillus, Penicillium, Trichoderma and Glioderma were preponderant fungus. All the fungal ITS rRNA gene sequences showed from 100 to 96% identity with the existing sequences of NCBI database (Additional file 1: Table S1) , These implied that different habitat of marine environment has rich marine fungi and showed abundant biological diversity.

### Antibacterial and antitumor activity of the fermentation product of marine fungi

Antibacterial and anticancer cell activity of marine fungi was shown in Additional file 2: Table S2. 17 strains showed no inhibitory effect on the four indicator bacteria or Melanoma cells B-16, accounting for 8.72% of the total isolates. The associations of inhibition effects and different habitats were presented in Table 2. The results also indicated that the marine fungi isolated from the three habitats have a wide range of biological activities.

**Table 2:** The associations between inhibition effects of marine fungi and different habitats

	*S. aureus*		*K. pneumoniae*		*P. aeruginosa*		*E. coli*		B-16	
	Strains	%	Strains	%	Strains	%	Strains	%	Strains	%
XB										
−	40	37.73	53	50.00	50	47.17	39	36.79	28	26.42
+	34	32.08	25	23.58	42	39.62	43	40.57	8	7.55
++	28	26.42	24	22.64	12	11.32	20	18.87	41	38.68
+++	4	3.77	4	3.77	2	1.89	4	3.77	29	27.35
NE										
−	13	36.11	23	63.89	16	44.44	19	52.78	10	27.78
+	15	41.67	10	27.78	17	47.22	13	36.11	5	13.89
++	6	16.67	1	2.78	3	8.33	1	2.78	20	55.56
+++	2	5.56	2	5.56	0	0	3	8.33	1	2.78
YM										
−	5	9.43	14	26.42	20	37.74	17	32.08	34	64.15
+	21	39.62	16	30.19	11	20.75	20	37.74	10	18.87
++	22	41.51	17	32.08	17	32.08	13	24.53	7	13.21
+++	5	9.43	6	11.32	5	9.43	3	5.66	2	3.77
Total										
−	58	29.74	90	46.15	86	44.1	75	38.46	72	36.92
+	70	35.9	51	26.15	70	35.9	76	38.97	23	11.79
++	56	28.82	42	21.54	32	16.41	34	17.44	68	34.87
+++	11	5.64	12	6.15	7	3.59	10	5.128	32	16.41

“−” bacteriostatic circle diameter <6 cm or IC_50_ > 500 μg/mL; “+” 6 cm ≤ this bacteriostatic circle diameter <10 cm or 250 μg/mL ≤ IC_50_ < 500 μg/mL; “++” 10 cm ≤ this bacteriostatic circle diameter < 15 cm or 100 μg/mL ≤ IC_50_? < 250 μg/mL; “+++” bacteriostatic circle diameter ≥15 cm or IC_50_ ≤ 100 μg/mL

**Fig. 3:**
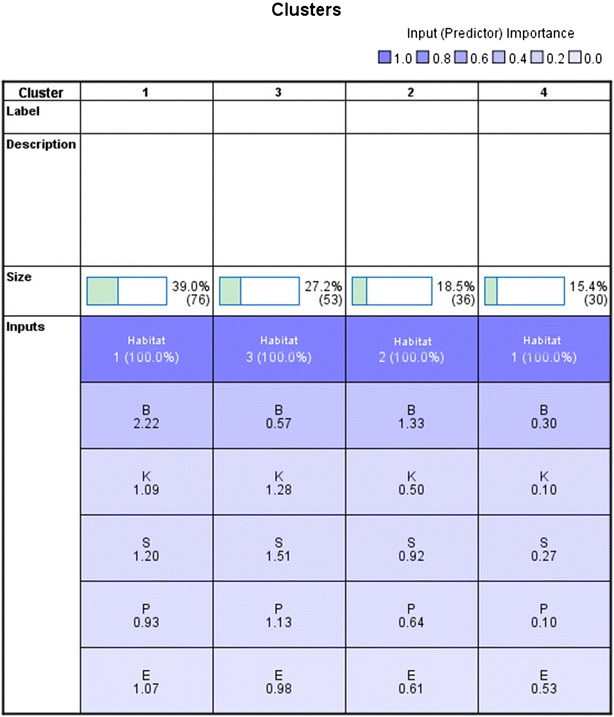
Clustering analysis of fungi with different biological activity fungi (B is Melanoma cells B-16, S is *S. aureus*, P is *P. aeruginosa*, K is *K. pneumoniae*, E is *E. coli*)

195 Fungi isolated from three habitats can be divided into 4 clusters on the basis of the inhibition acitivity of the pathogenic bacteria and tumor cells (Fig. 3). Additional file 3: Figure S1 and Table 2 showed that the fungi of the cluster 3 had strongest antibacterial activity on *S. aureus, P. aeruginosa, K. pneumoniae,* than those of other clusters. However, the strains of cluster 1 appeared to be more effective on the Melanoma cells B-16 and *E*. *coli* than those of other clusters. The results of Table 2 and Additional file 3: Figure S1 also indicated that the strains separated from breach habitat in West Coastal of Haikou had the strongest antitumor activity, while the isolations of the mangrove forest habitats had the weakest antitumor activity. Nevertheless the fungi isolated from the mangrove forest habitats exhibited the strongest antibacterial activity, while the isolations from the estuarine habitats of Nan Dujiang River showed the weakest antibacterial activity.

**Fig. 4:**
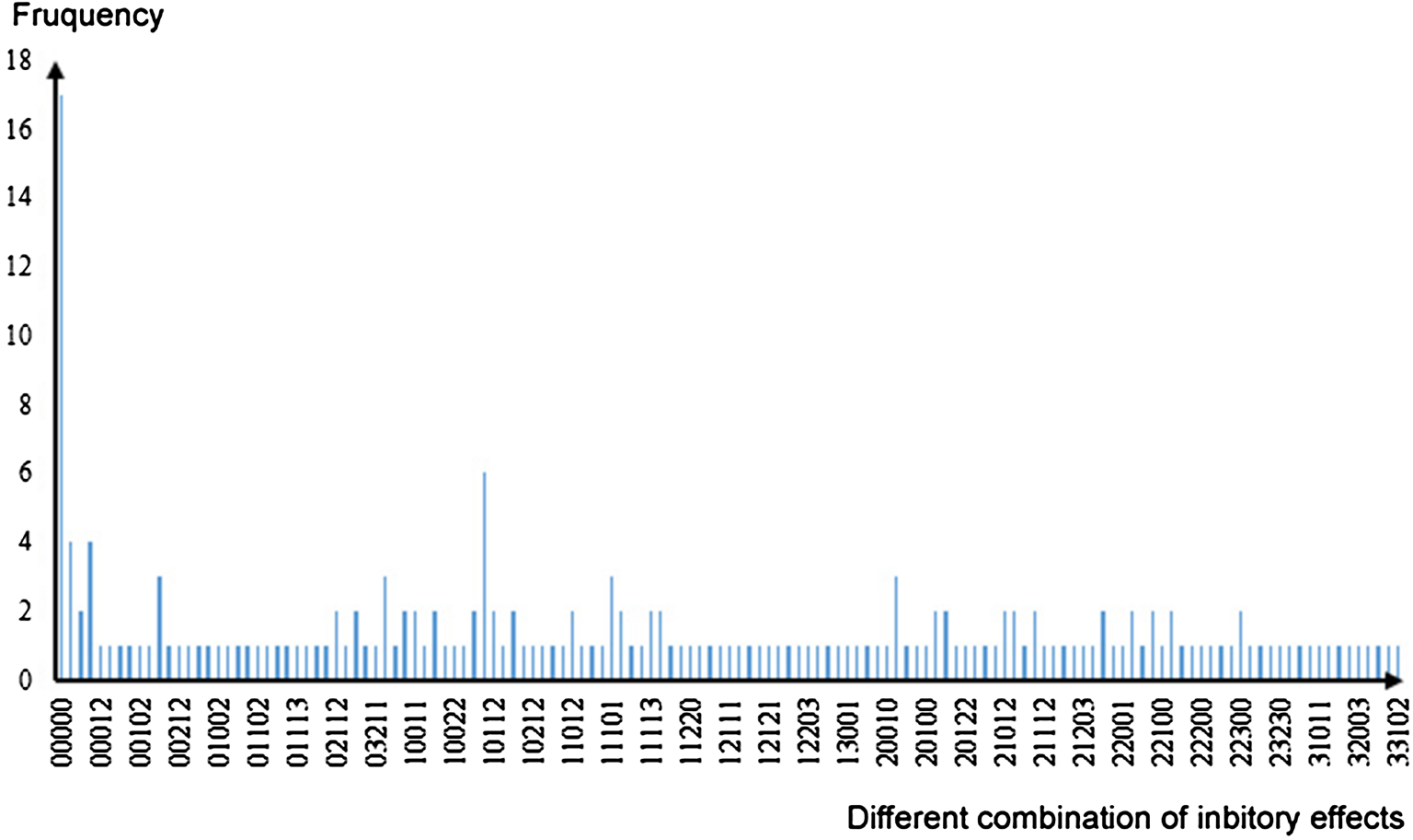
The frequency of different combination of inhibition activity

For further analysis, 137 combination results of inhibition activity were found (showed in Fig. 4). It means that 195 fungi can be classified into 137 groups with different biological activities. It is also suggested that the three different habitats are rich in species diversity of marine fungi.

## Discussion

Fungal biodiversity assessments are essential for exploring diversity from not only the biogeographical perspective, but also the view of bioactive secondary metabolites, which help to establish conservation policies Ding et al.(Ding et al. 2011; Duarte et al. 2012). Diversity of fungi has been studied from various habitats including seawater, coastal waters, deep-sea sediments, hypersaline waters, mangroves, salt marshes, and hydrothermal vents (Overy et al. 2014). As is known to all, the coastal region is a dynamic, self-sustained system with biodiversity, meanwhile different marine fungi produced various metabolites which have wide applications in pharmaceutics (Gerwick and Fenner 2013). The aim of this study was to investigate fungal diversity from various marine habitats. This study showed that the secondary metabolites produced by fungi in different coastal habitats had different strength biological activity.

Mangrove habitats are a type of intertidal wetland ecosystems, which are sensitive to global climate change. This is a semi open system, due to tides rise and fall, the environment changes frequently, mangrove plants are deciduous, so mangrove habitats contain abundant organic carbon which allows microbes to thrive inalo (Departamento et al. 2012; Azman et al. 2015). Mangrove sediments are characteristic of strong reduction, low pH, high salinity, nutrient rich, etc., which have rich resources in microbe (Andreote et al. 2012). K–B experiments showed that fungi isolation from the mangrove habitats had strongest antibacterial activity. The reason for this may be due to the intense competition between species, which evolved to secrete substances with high antibacterial activity inhibiting the growth of other strains. Zhang (2013) analyzed the literature on active ingredient of mangrove fungi in China, finding that there are nine compounds separated from fungi of mangrove forests in Hainan having antibacterial activity. This suggests that mangrove habitats are rich in fungi with bacteriostatic activity.

The MTT assay showed that the strains separated from beach habitats had the strongest antitumor activity. It is probable that along the west coast, there is no sewage flowing into the sea, which contained lots of harmful substances hazardous waste, include in the vicinity of the Haikou Port and new port, household garbage and other harmful substances accumulated in the habitats, which makes fungi adapt to the harsh environments.

## Conclusions

195 strains of marine fungi were isolated and purified from the three coastal habitats (i.e., beach habitats, estuarine habitats and mangrove habitats). K–B method and MTT method were applied to evaluate the antibacterial and antitumor cell activity for secondary metabolites of marine fungi. The experiment results showed that fungi isolation from the mangrove habitats had the strongest antibacterial activity, while strains separated from beach habitats had the strongest antitumor activity. Further analysis indicated that 195 fungal strains fell into 137 groups of different combination of inhibitory activity. This work may help to broaden our understanding of the fungal diversity in various marine habitats with different biological activity biological activity, and offer some useful experiences to select fungi for producing bioactive compounds from various marine habitats.

## Additional files

**Additional file 1: Table S1.** Phylogenetic affiliations of culturable fungi based on of ITS rDNA gene sequences.

**Additional file 2: Table S2.** The inhibitory effects of marine fungi on four strains of pathogenic bacteria and melanoma cells B-16.

**Additional file 3: Figure S1.** Phylogenetic tree based on ITS rRNA genes from three coastal habitats.
