# Supplementary material for: A study on biological activity of marine fungi from different habitats in coastal regions
Source: Springerplus. 2016 Nov 14;5(1):1966. doi: 10.1186/s40064-016-3658-3 (PMC5108748; doi:10.1186/s40064-016-3658-3)
Supplement: Supplementary file 1 — Additional file 1: Table S1. Phylogenetic affiliations of culturable fungi based on of ITS rDNA gene sequences. [file 40064_2016_3658_MOESM1_ESM.doc]

**Additional file 1:**

Table S1: Phylogenetic affiliations of culturable fungi based on of ITS rDNA gene sequences

| Isolate ID | GenBank accession No. | Closest identified relative | Class | Identity % |
| --- | --- | --- | --- | --- |
| XB-1 | WXEW16Y0015 | Colletotrichum kahawae KT375326.1 | Glomerellaceae | 99 |
| XB-2 | WXEW16Y0016 | Guignardia musicola NR_137716.1 | Botryosphaeriales | 99 |
| XB-3 | WXEW16Y0017 | Psiloglonium araucanum FJ161149.2 | Hysteriaceae | 100 |
| XB-4 | WXEW16Y0018 | Isaria javanica FJ765270.1 | Cordycipitaceae | 97 |
| XB-5 | WXEW16Y0019 | Zymoseptoria tritici KM852874.1 | Mycosphaerellaceae | 97 |
| XB-6 | WXEW16Y0020 | Aspergillus costaricaensis FJ629327.1 | Aspergillaceae | 99 |
| XB-7 | WXEW16Y0021 | Elsinoe fawcettii KF010913.1 | Elsinoaceae | 100 |
| XB-8 | WXEW16Y0022 | Hypocrella libera DQ070209.1 | Clavicipitaceae | 96 |
| XB-9 | WXEW16Y0023 | Ceratocystis norvegica NR_136978.1 | Ceratocystidacea | 99 |
| XB-10 | WXEW16Y0024 | Trichoderma neokoningii NR_138446.1 | Hypocreaceae | 100 |
| XB-11 | WXEW16Y0025 | Wrightella coccinea JX203858.1 | Melithaeidae | 98 |
| XB-12 | WXEW16Y0026 | Eremothecium sinecaudum AF442275.1 | Saccharomycetaceae | 97 |
| XB-13 | WXEW16Y0027 | Aspergillus fischeri NR_137479.1 | Aspergillaceae | 99 |
| XB-14 | WXEW16Y0028 | Gliocladium cibotii FJ537114.1 | Hypocreales | 99 |
| XB-15 | WXEW16Y0029 | Botrytis sinoviticola JN692383.1 | Sclerotiniaceae | 98 |
| XB-16 | WXEW16Y0030 | Penicillium buchwaldii JX313164.1 | Aspergillaceae | 99 |
| XB-17 | WXEW16Y0031 | Calospora arausiaca KP717079.1 | Melanconidaceae | 96 |
| XB-18 | WXEW16Y0032 | Uromyces trifolii-repentis DQ925302.1 | Pucciniaceae | 98 |
| XB-19 | WXEW16Y0033 | Tryblidiopsis pinastri JF793678.1 | Rhytismataceae | 99 |
| XB-20 | WXEW16Y0034 | Cercospora apiicola AY840440.1 | Mycosphaerellaceae | 100 |
| XB-21 | WXEW16Y0035 | Fabrella tsugae U92304.1 | Hemiphacidiaceae | 98 |
| XB-22 | WXEW16Y0036 | Penicillium sclerotiorum JX076979.1 | Aspergillaceae | 99 |
| XB-23 | WXEW16Y0037 | Chloroscypha chloromela U92311.1 | Helotiaceae | 99 |
| XB-24 | WXEW16Y0038 | Colpoma quercinum U92306.1 | Rhytismataceae | 100 |
| XB-25 | WXEW16Y0039 | Guignardia aesculi AB095504.1 | Phyllostictaceae | 98 |
| XB-26 | WXEW16Y0040 | Melia azedarach GU135144.1 | Pentapetalae | 98 |
| XB-27 | WXEW16Y0041 | Diaporthe sojae KJ659202.1 | Diaporthaceae | 97 |
| XB-28 | WXEW16Y0042 | Hysterium angustatum GU456341.1 | Hysteriaceae | 99 |
| XB-29 | WXEW16Y0043 | Colletotrichum gloeosporioides AF264032.1 | Glomerellaceae | 96 |
| XB-30 | WXEW16Y0044 | Polycoccum vermicularium AY961601.1 | Polycoccaceae | 100 |
| XB-31 | WXEW16Y0045 | Candida albicans BD309288.1 | Debaryomycetaceae | 99 |
| XB-32 | WXEW16Y0046 | XB-50 Boeremia exigua KR653200.1 | Pleosporineae | 96 |
| XB-33 | WXEW16Y0047 | Eremothecium ashbyi AB294412.1 | Saccharomycetaceae | 98 |
| XB-34 | WXEW16Y0048 | Tryblidiopsis pinastri JF793678.1 | Rhytismataceae | 99 |
| XB-35 | WXEW16Y0049 | Rosenscheldiella brachyglottidis GQ355335.1 | Mycosphaerellaceae | 97 |
| XB-36 | WXEW16Y0050 | Bacillus subtilis KN049967.1 | Bacillaceae | 99 |
| XB-37 | WXEW16Y0051 | Scopulariopsis brevicaulis FJ025211.1 | Microascaceae | 100 |
| XB-38 | WXEW16Y0052 | Zymoseptoria tritici KM852874.1 | Mycosphaerellaceae | 100 |
| XB-39 | WXEW16Y0053 | Melanconium elaeidicola KR476788.1 | Dothioraceae | 99 |
| XB-40 | WXEW16Y0054 | Penicillium mallochii JN626113.1 | Aspergillaceae | 98 |
| XB-41 | WXEW16Y0055 | Darkera parca KM108381.1 | Darkera | 97 |
| XB-42 | WXEW16Y0056 | Gremmenia infestans KM216393.1 | Phacidiaceae | 100 |
| XB-43 | WXEW16Y0057 | XB-50 Boeremia exigua KR653200.1 | Pleosporineae | 99 |
| XB-44 | WXEW16Y0058 | Trichoderma viride FJ904855.1 | Hypocreaceae | 98 |
| XB-45 | WXEW16Y0059 | Aspergillus lentulus AY738521.1 | Aspergillaceae | 99 |
| XB-46 | WXEW16Y0060 | Fusarium verticillioides JX971228.1 | Nectriaceae | 97 |
| XB-47 | WXEW16Y0061 | Petriella sordid KP067263.1 | Microascaceae | 100 |
| XB-48 | WXEW16Y0062 | Thanatephorus cucumeris EU244845.1 | Ceratobasidiaceae | 100 |
| XB-49 | WXEW16Y0063 | Uromyces viciae-fabae AF426199.1 | Pucciniaceae | 99 |
| XB-50 | WXEW16Y0064 | Anthomastus ritteri JX203824.1 | Alcyoniidae | 100 |
| XB-51 | WXEW16Y0065 | Hysterium vermiforme GQ221929.1 | Hysteriaceae | 98 |
| XB-52 | WXEW16Y0066 | Marssonia populi EU622260.1 | Mitosporic Ascomycota | 98 |
| XB-53 | WXEW16Y0067 | Phaeoseptoria musae GQ179756.1 | Mitosporic Ascomycota | 99 |
| XB-54 | WXEW16Y0068 | Diaporthe sojae KJ659202.1 | Diaporthaceae | 96 |
| XB-55 | WXEW16Y0069 | Meria laricis U92298.1 | Rhytismataceae | 98 |
| XB-56 | WXEW16Y0070 | Elsinoe fawcettii KF010913.1 | Elsinoaceae | 99 |
| XB-57 | WXEW16Y0071 | Hysterium vermiforme GQ221929.1 | Hysteriaceae | 99 |
| XB-58 | WXEW16Y0072 | Botryosphaeria corticis DQ299246.1 | Botryosphaeriaceae | 99 |
| XB-59 | WXEW16Y0073 | Trichoderma viride FJ904855.1 | Hypocreaceae | 97 |
| XB-60 | WXEW16Y0074 | Physalospora zeicola DQ377881.1 | Hyponectriaceae | 99 |
| XB-61 | WXEW16Y0075 | Cephalosporium gramineum HQ322368.1 | Hypocreales | 98 |
| XB-62 | WXEW16Y0076 | Gliocladium deliquescens GQ229478.1 | Hypocreales | 100 |
| XB-63 | WXEW16Y0077 | Macrophoma sugi AB541379.1 | Botryosphaeriaceae | 100 |
| XB-64 | WXEW16Y0078 | Pezicula sporulosa KR859265.1 | Dermateaceae | 99 |
| XB-65 | WXEW16Y0079 | Chaetomium longicolleum KT149498.1 | Chaetomiaceae | 97 |
| XB-66 | WXEW16Y0080 | Colletotrichum limetticola KM251962.1 | Glomerellaceae | 99 |
| XB-67 | WXEW16Y0081 | Gliocladium deliquescens GQ229478.1 | Hypocreales | 98 |
| XB-68 | WXEW16Y0082 | Eremothecium coryli AB478307.1 | Saccharomycetaceae | 99 |
| XB-69 | WXEW16Y0083 | Marssonia populi EU622260.1 | Mitosporic Ascomycota | 99 |
| XB-70 | WXEW16Y0084 | Gliocladium deliquescens GQ229478.1 | Hypocreales | 98 |
| XB-71 | WXEW16Y0085 | Botrytis mali EF216694.1 | Sclerotiniaceae | 99 |
| XB-72 | WXEW16Y0086 | Marssonina rosae FJ493249. | Dermateaceae | 100 |
| XB-73 | WXEW16Y0087 | Gliocladium deliquescens GQ229478.1 | Hypocreales | 100 |
| XB-74 | WXEW16Y0088 | Trichoderma sinensis GU176476.1 | Hypocreaceae | 99 |
| XB-75 | WXEW16Y0089 | Myceliophthora thermophila KT287076.1 | Chaetomiaceae | 97 |
| XB-76 | WXEW16Y0090 | Sphaceloma tectificae GQ303325.1 | Mitosporic Ascomycota | 99 |
| XB-77 | WXEW16Y0091 | Uromyces trifolii-repentis DQ925302.1 | Pucciniaceae | 97 |
| XB-78 | WXEW16Y0092 | Guignardia cryptomeriae KF941276.1 | Botryosphaeriales | 100 |
| XB-79 | WXEW16Y0093 | Pythium ultimum KU672382.1 | Pythiaceae | 99 |
| XB-80 | WXEW16Y0094 | Gremmenia infestans KM216393.1 | Phacidiaceae | 99 |
| XB-81 | WXEW16Y0095 | Sporotrichum columbiense EU427035.1 | Agaricomycotina | 100 |
| XB-82 | WXEW16Y0096 | Darkera parca KM108381.1 | Darkera | 99 |
| XB-83 | WXEW16Y0097 | Glonium stellatum KV748463.1 | Gloniaceae | 97 |
| XB-84 | WXEW16Y0098 | Paecilomyces dactylethromorphus KF530866.1 | Thermoascaceae | 99 |
| XB-85 | WXEW16Y0099 | Coniochaeta tetraspora AF048813.1 | Coniochaetaceae | 97 |
| XB-86 | WXEW16Y0100 | Tranzschelia pruni-spinosae DQ363329.1 | Uropyxidaceae | 98 |
| XB-87 | WXEW16Y0101 | Lophotrichus plumbescens AF048805.1 | Microascaceae | 99 |
| XB-88 | WXEW16Y0102 | Wrightella coccinea JX203858.1 | Melithaeidae | 97 |
| XB-89 | WXEW16Y0103 | Sphaceloma protearum AF131084.1 | Mitosporic Ascomycota | 99 |
| XB-90 | WXEW16Y0104 | Penicillium viticola JN686439.1 | Aspergillaceae | 96 |
| XB-91 | WXEW16Y0105 | Cephalosporium caerulens AY566994.1 | Hyohomycetaceae | 98 |
| XB-92 | WXEW16Y0106 | Lophodermium australe KM117224.1 | Rhytismataceae | 99 |
| XB-93 | WXEW16Y0107 | Barriopsis fusca EU673109.1 | Botryosphaeriaceae | 99 |
| XB-94 | WXEW16Y0108 | Septogloeum mori HQ339998.1 | Mitosporic Ascomycota | 100 |
| XB-95 | WXEW16Y0109 | Exophiala castellanii AB025831.1 | Herpotrichiellaceae | 100 |
| XB-96 | WXEW16Y0110 | Helminthosporium solani KC106739.1 | Mitosporic Ascomycota | 98 |
| XB-97 | WXEW16Y0111 | Puccinia psidii KQ003388.1 | Pucciniaceae | 98 |
| XB-98 | WXEW16Y0112 | Anthomastus ritteri JX203824.1 | Alcyoniidae | 100 |
| XB-99 | WXEW16Y0113 | Botryosphaeria corticis DQ299246.1 | Botryosphaeriaceae | 100 |
| XB-100 | WXEW16Y0114 | Xylogone sphaerospora JF681944.1 | Leotiomycetes | 99 |
| XB-101 | WXEW16Y0115 | Fusarium decemcellulare AH001643.2 | Nectriaceae | 98 |
| XB-102 | WXEW16Y0116 | Trichoderma viride FJ904855.1 | Hypocreaceae | 99 |
| XB-103 | WXEW16Y0117 | Anthomastus ritteri JX203824.1 | Alcyoniidae | 98 |
| XB-104 | WXEW16Y0118 | Botryosporium longibrachiatum KF372591.1 | Mitosporic Ascomycota | 99 |
| XB-105 | WXEW16Y0119 | Lophodermium australe KM117224.1 | Rhytismataceae | 97 |
| XB-106 | WXEW16Y0120 | Guignardia cryptomeriae KF941276.1 | Botryosphaeriales | 98 |
| NE-1 | WYPSD7R0015 | Elsinoe fawcettii KF010913.1 | Elsinoaceae | 99 |
| NE-2 | WYPSD7R0016 | Fusarium decemcellulare AH001643.2 | Nectriaceae | 100 |
| NE-3 | WYPSD7R0017 | Fusarium decemcellulare AH001643.2 | Nectriaceae | 99 |
| NE-4 | WYPSD7R0018 | Darkera parca KM108381.1 | Darkera | 98 |
| NE-5 | WYPSD7R0019 | Aschersonia tahitensis EF190308.1 | Clavicipitaceae | 100 |
| NE-6 | WYPSD7R0020 | Xylaria nigripes KR534724.1 | Xylariaceae | 99 |
| NE-7 | WYPSD7R0021 | Elsinoe fawcettii KF010913.1 | Elsinoaceae | 99 |
| NE-8 | WYPSD7R0022 | Marssonina rosae FJ493249.1 | Dermateaceae | 100 |
| NE-9 | WYPSD7R0023 | Marssonina brunnea KM246343.1 | Dermateaceae | 98 |
| NE-10 | WYPSD7R0024 | Botrytis mali EF216694.1 | Sclerotiniaceae | 99 |
| NE-11 | WYPSD7R0025 | Physalospora scirpi KF871446.1 | Hyponectriaceae | 100 |
| NE-12 | WYPSD7R0026 | Septogloeum sojae JX853748.1 | Mitosporic Ascomycota | 99 |
| NE-13 | WYPSD7R0027 | Darkera parca KM108381.1 | Darkera | 100 |
| NE-14 | WYPSD7R0028 | Botryosporium longibrachiatum KF372591.1 | Mitosporic Ascomycota | 99 |
| NE-15 | WYPSD7R0029 | Trichoderma turrialbense EU330944.1 | Hypocreaceae | 100 |
| NE-16 | WYPSD7R0030 | Penicillium buchwaldii JX313164.1 | Aspergillaceae | 98 |
| NE-17 | WYPSD7R0031 | Aschersonia tahitensis EF190308.1 | Clavicipitaceae | 97 |
| NE-18 | WYPSD7R0032 | Phyllosticta citriasiana FJ538414.1 | Phyllostictaceae | 97 |
| NE-19 | WYPSD7R0033 | Aspergillus oryzae EU680476.1 | Aspergillaceae | 98 |
| NE-20 | WYPSD7R0034 | Monochaetia camelliae AY682948.1 | Amphisphaeriaceae | 99 |
| NE-21 | WYPSD7R0035 | Pestalotia vaccinii DQ787844.1 | Amphisphaeriaceae | 99 |
| NE-22 | WYPSD7R0036 | Aspergillus costaricaensis FJ629327.1 | Aspergillaceae | 98 |
| NE-23 | WYPSD7R0037 | Hysterium angustatum GU456341.1 | Hysteriaceae | 100 |
| NE-24 | WYPSD7R0038 | Dothichiza pithyophila FJ150969.1 | Dothioraceae | 99 |
| NE-25 | WYPSD7R0039 | Physalospora vaccinii FJ603608.1 | Phyllostictaceae | 100 |
| NE-26 | WYPSD7R0040 | Cespitularia erecta JX203869.1 | Xeniidae | 99 |
| NE-27 | WYPSD7R0041 | Marssonina brunnea KM246343.1 | Dermateaceae | 98 |
| NE-28 | WYPSD7R0042 | Fusarium verticillioides JX971228.1 | Nectriaceae | 99 |
| NE-29 | WYPSD7R0043 | Cephalosporium humicola JQ724518.1 | Hyohomycetaceae | 97 |
| NE-30 | WYPSD7R0044 | Gloeosporium ampelophagum JN122426.1 | Pezizomycotina incertae | 99 |
| NE-31 | WYPSD7R0045 | Cassidula cf. labrella HQ660016.1 | Ellobioidea | 100 |
| NE-32 | WYPSD7R0046 | Penicillium spathulatum JX313158.1 | Aspergillaceae | 99 |
| NE-33 | WYPSD7R0047 | Monochaetia kansensis DQ534047.1 | Amphisphaeriaceae | 100 |
| NE-34 | WYPSD7R0048 | Trichoderma viride AY380909.1 | Hypocreaceae | 99 |
| NE-35 | WYPSD7R0049 | Trichoderma viride FJ904855.1 | Hypocreaceae | 99 |
| NE-36 | WYPSD7R0050 | Glonium stellatum KV748445 | Pleosporomycetidae | 98 |
| YM-1 | WYPSD7R0015 | Truncatella betulae DQ278920.1 | Sporocadaceae | 99 |
| YM-2 | WYPSD7R0051 | Achaetomium strumarium KT371346.1 | Chaetomiaceae | 97 |
| YM-3 | WYPSD7R0052 | Penicillium buchwaldii JX313164.1 | Aspergillaceae | 99 |
| YM-4 | WYPSD7R0053 | Trichoderma longibrachiatum AJ867242.1 | Hypocreaceae | 96 |
| YM-5 | WYPSD7R0054 | Pestalotiopsis psidii GU905995.1 | Amphisphaeriaceae | 99 |
| YM-6 | WYPSD7R0055 | Achaetomium strumarium KT371346.1 | Chaetomiaceae | 98 |
| YM-7 | WYPSD7R0056 | Verticillium longisporum HQ414941.2 | Plectosphaerellaceae | 99 |
| YM-8 | WYPSD7R0057 | Penicillium oxalicum HQ703581.1 | Aspergillaceae | 98 |
| YM-9 | WYPSD7R0058 | Diaporthe foeniculina KX056244.1 | Diaporthaceae | 99 |
| YM-10 | WYPSD7R0059 | Aspergillus oryzae EU680476.1 | Aspergillaceae | 97 |
| YM-11 | WYPSD7R0060 | Marssonina rosae AY904059.1 | Dermateaceae | 99 |
| YM-12 | WYPSD7R0061 | Wrightella coccinea JX203858.1 | Melithaeidae | 98 |
| YM-13 | WYPSD7R0062 | Cephalosporium gramineum HQ322368.1 | Hypocreales | 97 |
| YM-14 | WYPSD7R0063 | Kernia nitida KC485065.1 | Microascaceae | 99 |
| YM-15 | WYPSD7R0064 | Phanerochaete chrysosporium AY442335.1 | Mitosporic Ascomycota | 99 |
| YM-16 | WYPSD7R0065 | Cephalosporium gramineum HQ322368.1 | Hypocreales | 98 |
| YM-17 | WYPSD7R0066 | Zymoseptoria tritici KM852874.1 | Mycosphaerellaceae | 99 |
| YM-18 | WYPSD7R0067 | Colletotrichum limetticola KM251962.1 | Glomerellaceae | 98 |
| YM-19 | WYPSD7R0068 | Gloeosporium ampelophagum JN122426.1 | Pezizomycotina incertae | 100 |
| YM-20 | WYPSD7R0069 | Kernia nitida KC485065.1 | Microascaceae | 100 |
| YM-21 | WYPSD7R0070 | Ceratocystis eucalypticola FJ236783.1 | Ceratocystidaceae | 99 |
| YM-22 | WYPSD7R0071 | Melanconium elaeidicola KR476788.1 | Dothioraceae | 100 |
| YM-23 | WYPSD7R0072 | Cornularia cornucopiae JX203848.1 | Cornulariidae | 98 |
| YM-24 | WYPSD7R0073 | Melanconium hedericola KP004461.1 | Dothioraceae | 99 |
| YM-25 | WYPSD7R0074 | Penicillium jacksonii JN686438.1 | Aspergillaceae | 97 |
| YM-26 | WYPSD7R0075 | Monilia mumecola AB125614.1 | Sclerotiniaceae | 99 |
| YM-27 | WYPSD7R0076 | Lophodermium austral KM117224.1 | Rhytismataceae | 98 |
| YM-28 | WYPSD7R0077 | Sporotrichum roseum JQ724438.1 | Agaricomycotina | 99 |
| YM-29 | WYPSD7R0078 | Ascotricha xylina AF048789.1 | Xylariaceae | 100 |
| YM-30 | WYPSD7R0079 | Diaporthe longicolla HM347700.1 | Diaporthaceae | 99 |
| YM-31 | WYPSD7R0080 | Chaetomium cucumericola KT214757.1 | Chaetomiaceae | 97 |
| YM-32 | WYPSD7R0081 | Metasphaeria deviate JN606418.1 | Dothioraceae | 96 |
| YM-33 | WYPSD7R0082 | Lophotrichus plumbescens AF048805.1 | Microascaceae | 99 |
| YM-34 | WYPSD7R0083 | Monilia fructigena EU622274.1 | Sclerotiniaceae | 99 |
| YM-35 | WYPSD7R0084 | Chaetomium subfunicola GU563368.1 | Chaetomiaceae | 98 |
| YM-36 | WYPSD7R0085 | Rhabdocline pseudotsugae KP001552.1 | Hemiphacidiaceae | 98 |
| YM-37 | WYPSD7R0086 | Botrytis sinoallii FJ169664.2 | Sclerotiniaceae | 100 |
| YM-38 | WYPSD7R0087 | Puccinia hysterium DQ925270.1 | Pucciniaceae | 99 |
| YM-39 | WYPSD7R0088 | Guignardia aesculi AB095504.1 | Phyllostictaceae | 97 |
| YM-40 | WYPSD7R0089 | Melanconium hedericola KP004461.1 | Dothioraceae | 99 |
| YM-41 | WYPSD7R0090 | Aspergillus flavus JX502763.1 | Aspergillaceae | 98 |
| YM-42 | WYPSD7R0091 | Candida tropicalis AY170877.1 | Saccharomycetaceae | 97 |
| YM-43 | WYPSD7R0092 | Ceratocystis harringtonii KR347459.1 | Ceratocystidaceae | 99 |
| YM-44 | WYPSD7R0093 | Scopulariopsis hibernica KC460812.1 | Microascaceae | 99 |
| YM-45 | WYPSD7R0094 | Chaetomium truncatulum KT149485.1 | Chaetomiaceae | 99 |
| YM-46 | WYPSD7R0095 | Cephalosporium humicola JQ724518.1 | Hypocreales | 99 |
| YM-47 | WYPSD7R0096 | Glonium stellatum KV748463.1 | Gloniaceae | 98 |
| YM-48 | WYPSD7R0097 | Rhabdocline weirii AF260814.1 | Hemiphacidiaceae | 99 |
| YM-49 | WYPSD7R0098 | Penicillium glabrum FJ618520.1 | Aspergillaceae | 97 |
| YM-50 | WYPSD7R0099 | Stachybotrys elegans EU008754.1 | Stachybotriaceae | 100 |
| YM-51 | WYPSD7R0100 | Trichoderma asperelloides GU248412.1 | Hypocreaceae | 99 |
| YM-52 | WYPSD7R0101 | Scopulariopsis murina KF986441.1 | Microascaceae | 98 |
| YM-53 | WYPSD7R0102 | Cyclaneusma minus U92309.1 | Helotiales | 99 |
