# Supplementary material for: A study on biological activity of marine fungi from different habitats in coastal regions
Source: Springerplus. 2016 Nov 14;5(1):1966. doi: 10.1186/s40064-016-3658-3 (PMC5108748; doi:10.1186/s40064-016-3658-3)
Supplement: Supplementary file 2 — Additional file 2: Table S2. The inhibitory effects of marine fungi on four strains of pathogenic bacteria and melanoma cells B-16. [file 40064_2016_3658_MOESM2_ESM.doc]

**Additional file 2:**

**Table S2** The inhibitory effects of marine fungi on four strains of pathogenic bacteria and melanoma cells B-16

| Strains.  No. | *S. aureus* | *K .pneu moniae* | *P.aeru ginosa* | *E. coli* | *B-16* | Strains  No. | *S. aureus* | *K.pne umoniae* | *P.aeru ginosa* | *E. coli* | *B-16* |
| --- | --- | --- | --- | --- | --- | --- | --- | --- | --- | --- | --- |
| XB-1 | + | - | + | + | ++ | XB-99 | + | - | - | ++ | + |
| XB-2 | ++ | ++ | - | ++ | +++ | XB-100 | ++ | ++ | ++ | ++ | - |
| XB-3 | ++ | - | ++ | ++ | + | XB-101 | - | - | - | - | - |
| XB-4 | + | - | ++ | + | ++ | XB-102 | - | - | - | - | - |
| XB-5 | + | + | + | + | ++ | XB-103 | + | + | - | + | ++ |
| XB-6 | + | - | + | ++ | +++ | XB-104 | - | - | - | - | ++ |
| XB-7 | + | - | + | - | ++ | XB-105 | + | + | +++ | +++ | +++ |
| XB-8 | ++ | - | + | ++ | - | XB-106 | - | ++ | ++ | + | +++ |
| XB-9 | + | - | + | - | - | NE-1 | + | - | + | - | ++ |
| XB-10 | + | + | + | - | ++ | NE-2 | - | - | - | - | - |
| XB-11 | + | + | + | - | - | NE-3 | - | - | - | - | - |
| XB-12 | + | ++ | + | ++ | ++ | NE-4 | - | - | - | - | - |
| XB-13 | ++ | +++ | - | ++ | +++ | NE-5 | ++ | + | - | + | ++ |
| XB-14 | - | ++ | - | ++ | ++ | NE-6 | - | - | ++ | + | ++ |
| XB-15 | - | - | + | + | ++ | NE-7 | + | - | + | - | ++ |
| XB-16 | - | - | + | + | ++ | NE-8 | + | - | - | - | - |
| XB-17 | ++ | - | + | + | - | NE-9 | - | + | + | + | + |
| XB-18 | - | ++ | + | + | ++ | NE-10 | - | - | + | - | ++ |
| XB-19 | ++ | ++ | + | - | ++ | NE-11 | + | - | - | - | + |
| XB-20 | ++ | ++ | - | ++ | +++ | NE-12 | + | - | - | + | ++ |
| XB-21 | + | + | + | + | +++ | NE-13 | - | - | - | - | ++ |
| XB-22 | +++ | ++ | ++ | ++ | ++ | NE-14 | - | - | - | - | ++ |
| XB-23 | - | + | + | + | +++ | NE-15 | ++ | + | + | + | ++ |
| XB-24 | + | - | - | ++ | +++ | NE-16 | +++ | + | + | +++ | - |
| XB-25 | +++ | ++ | + | + | +++ | NE-17 | ++ | + | - | + | ++ |
| XB-26 | +++ | +++ | - | ++ | +++ | NE-18 | + | - | ++ | + | + |
| XB-27 | ++ | + | - | + | +++ | NE-19 | ++ | - | + | + | ++ |
| XB-28 | + | ++ | + | ++ | + | NE-20 | + | + | + | ++ | - |
| XB-29 | + | - | + | + | +++ | NE-21 | + | +++ | + | - | ++ |
| XB-30 | + | + | + | + | + | NE-22 | + | + | + | - | ++ |
| XB-31 | + | + | + | - | + | NE-23 | ++ | - | - | + | ++ |
| XB-32 | - | - | - | - | - | NE-24 | - | - | - | - | - |
| XB-33 | + | + | - | ++ | +++ | NE-25 | + | + | + | - | + |
| XB-34 | ++ | ++ | + | - | ++ | NE-26 | + | - | - | ++ | ++ |
| XB-35 | + | ++ | + | + | +++ | NE-27 | - | - | + | + | ++ |
| XB-36 | ++ | ++ | + | ++ | ++ | NE-28 | + | + | - | - | ++ |
| XB-37 | + | ++ | ++ | - | +++ | NE-29 | + | - | + | - | ++ |
| XB-38 | + | ++ | + | + | + | NE-30 | - | - | + | + | - |
| XB-39 | + | ++ | + | +++ | +++ | NE-31 | + | + | + | + | +++ |
| XB-40 | ++ | +++ | ++ | - | +++ | NE-32 | +++ | +++ | + | - | ++ |
| XB-41 | - | - | - | - | - | NE-33 | - | ++ | - | ++ | + |
| XB-42 | - | - | - | - | - | NE-34 | + | - | + | - | ++ |
| XB-43 | - | - | - | - | - | NE-35 | - | - | - | - | - |
| XB-44 | - | - | - | - | - | NE-36 | ++ | - | ++ | + | - |
| XB-45 | - | - | - | ++ | - | YM-1 | + | ++ | + | + | - |
| XB-46 | ++ | ++ | ++ | +++ | + | YM-2 | ++ | + | ++ | + | ++ |
| XB-47 | - | +++ | ++ | - | ++ | YM-3 | + | + | ++ | - | - |
| XB-48 | - | - | - | ++ | + | YM-4 | + | + | - | + | - |
| XB-49 | - | + | + | + | - | YM-5 | +++ | - | - | +++ | + |
| XB-50 | + | - | - | + | +++ | YM-6 | ++ | + | ++ | + | ++ |
| XB-51 | + | + | - | + | ++ | YM-7 | ++ | - | + | - | - |
| XB-52 | - | - | - | + | ++ | YM-8 | ++ | +++ | - | ++ | - |
| XB-53 | ++ | - | - | + | ++ | YM-9 | + | - | + | + | ++ |
| XB-54 | ++ | + | - | + | +++ | YM-10 | ++ | - | + | + | ++ |
| XB-55 | - | + | - | - | ++ | YM-11 | + | - | - | - | - |
| XB-56 | + | - | + | - | ++ | YM-12 | + | - | - | + | + |
| XB-57 | - | + | - | + | - | YM-13 | ++ | ++ | ++ | - | - |
| XB-58 | ++ | + | + | - | - | YM-14 | + | ++ | + | ++ | - |
| XB-59 | - | - | - | - | - | YM-15 | +++ | ++ | - | - | +++ |
| XB-60 | ++ | - | + | ++ | ++ | YM-16 | ++ | ++ | - | - | ++ |
| XB-61 | ++ | ++ | - | - | ++ | YM-17 | + | + | ++ | + | + |
| XB-62 | - | - | - | + | - | YM-18 | + | + | + | ++ | - |
| XB-63 | ++ | ++ | + | + | ++ | YM-19 | +++ | - | - | - | - |
| XB-64 | + | ++ | ++ | - | ++ | YM-20 | - | +++ | ++ | + | + |
| XB-65 | ++ | + | + | - | ++ | YM-21 | ++ | - | - | ++ | - |
| XB-66 | ++ | + | + | - | ++ | YM-22 | ++ | ++ | - | ++ | - |
| XB-67 | - | - | - | + | - | YM-23 | ++ | ++ | + | - | - |
| XB-68 | - | + | + | + | ++ | YM-24 | - | + | - | - | - |
| XB-69 | - | - | - | - | - | YM-25 | - | ++ | - | + | - |
| XB-70 | - | - | - | + | - | YM-26 | + | - | ++ | ++ | - |
| XB-71 | ++ | - | - | + | - | YM-27 | ++ | + | ++ | ++ | - |
| XB-72 | + | - | - | - | - | YM-28 | + | ++ | +++ | - | - |
| XB-73 | - | - | - | + | - | YM-29 | ++ | + | ++ | + | - |
| XB-74 | +++ | + | + | ++ | ++ | YM-30 | + | +++ | - | - | + |
| XB-75 | + | ++ | + | + | ++ | YM-31 | ++ | +++ | +++ | ++ | - |
| XB-76 | + | - | - | + | - | YM-32 | + | ++ | ++ | ++ | - |
| XB-77 | - | ++ | + | + | ++ | YM-33 | + | ++ | +++ | - | + |
| XB-78 | - | ++ | ++ | + | +++ | YM-34 | ++ | + | ++ | - | - |
| XB-79 | - | + | + | - | ++ | YM-35 | + | ++ | - | + | - |
| XB-80 | - | - | - | - | - | YM-36 | + | + | + | - | + |
| XB-81 | - | - | - | + | +++ | YM-37 | ++ | - | - | + | +++ |
| XB-82 | - | - | - | - | ++ | YM-38 | +++ | + | ++ | ++ | - |
| XB-83 | ++ | + | ++ | - | +++ | YM-39 | + | - | - | + | - |
| XB-84 | - | - | ++ | + | +++ | YM-40 | ++ | ++ | - | - | + |
| XB-85 | ++ | - | - | - | +++ | YM-41 | + | + | ++ | ++ | - |
| XB-86 | - | + | - | + | ++ | YM-42 | ++ | +++ | ++ | +++ | - |
| XB-87 | ++ | - | - | + | ++ | YM-43 | ++ | ++ | +++ | - | - |
| XB-88 | + | - | - | + | + | YM-44 | - | ++ | + | ++ | + |
| XB-89 | ++ | + | + | + | +++ | YM-45 | ++ | - | ++ | - | - |
| XB-90 | - | - | - | - | +++ | YM-46 | + | +++ | ++ | + | ++ |
| XB-91 | - | - | - | - | +++ | YM-47 | +++ | + | - | + | + |
| XB-92 | - | - | - | - | - | YM-48 | ++ | ++ | +++ | - | - |
| XB-93 | - | - | + | + | +++ | YM-49 | - | + | - | ++ | - |
| XB-94 | - | - | - | - | - | YM-50 | + | - | + | - | - |
| XB-95 | - | - | +++ | - | ++ | YM-51 | ++ | ++ | ++ | + | ++ |
| XB-96 | ++ | + | + | +++ | ++ | YM-52 | + | + | - | ++ | - |
| XB-97 | + | - | + | ++ | +++ | YM-53 | ++ | - | + | + | - |
| XB-98 | + | - | - | + | +++ | acetone | - | - | - | - | - |

Note: “ - ”: bacteriostatic circle diameter ＜6 mm or IC_50_>500 μg/mL; “ + ”: 6 mm ≤bacteriostatic circle diameter ＜10 mm or 250 μg/mL≤ IC_50_ <500 μg/mL; “ ++ ”: 10 mm≤bacteriostatic circle diameter＜15 mm or 100 μg/mL ≤ C_50_ <250 μg/mL；“ +++ ”: bacteriostatic circle diameter ≥15 mm or IC50 ≤ 100μg/mL.
