# Supplementary figures and images for: A study on biological activity of marine fungi from different habitats in coastal regions
Source: Springerplus. 2016 Nov 14;5(1):1966. doi: 10.1186/s40064-016-3658-3 (PMC5108748; doi:10.1186/s40064-016-3658-3)

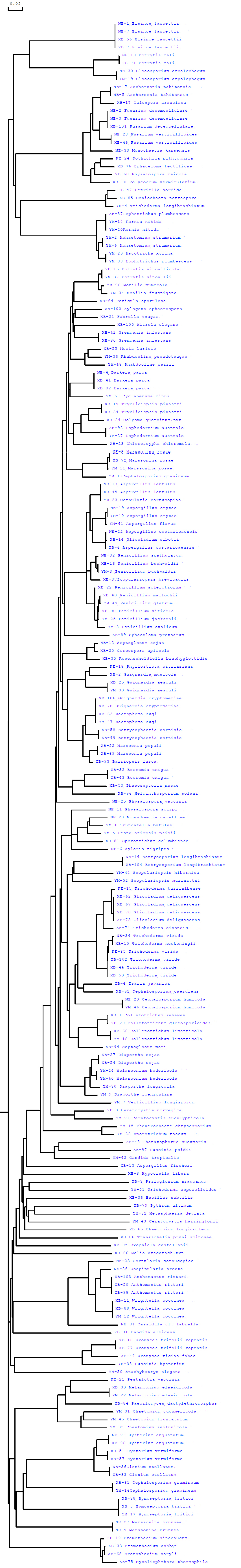

Supplement: Supplementary file 3 — Additional file 3: Figure S1. Phylogenetic tree based on ITS rRNA genes from three coastal habitats. [file 40064_2016_3658_MOESM3_ESM.jpg]
